# 2365. Fostering the Future: Improving Infection Prevention and Control Education in Pediatric Infectious Diseases Fellowship

**DOI:** 10.1093/ofid/ofac492.172

**Published:** 2022-12-15

**Authors:** Yasaman Fatemi, Marisse Plaras, Lauren Le Goff, Lori Handy, Sarah Smathers, Julia S Sammons, Susan Coffin, Eimear Kitt

**Affiliations:** Seattle Children's Hospital, Seattle, WA; Children's Hospital of Philadelphia, Philadelphia, Pennsylvania; Children's Hospital of Philadelphia, Philadelphia, Pennsylvania; Children's Hospital of Philadelphia, Philadelphia, Pennsylvania; Children's Hospital of Philadelphia, Philadelphia, Pennsylvania; Children's Hospital of Philadelphia, Philadelphia, Pennsylvania; Children's Hospital of Philadelphia, Philadelphia, Pennsylvania; Children's Hospital of Philadelphia, Philadelphia, Pennsylvania

## Abstract

**Background:**

Infection prevention and control (IPC) is a potential area of career specialization for infectious diseases (ID) fellows. However, ID fellows are not consistently involved in IPC operations or content expertise.

IPC education for ID fellows at Children’s Hospital of Philadelphia relied upon lectures, self-study, and some in-person shadowing. ID fellows had few experiences engaging in surveillance, working with data, or discussing approaches to common clinical IPC scenarios.

We aimed to develop an IPC curriculum for pediatric ID fellows focusing on experiential learning relevant to clinical and operational practice as an ID physician.

**Methods:**

We used the Kern model to systematically design a curriculum addressing identified gaps in IPC education. Areas of need included: aligning IPC curriculum content with the physician role, improving tracking of core IPC experiences (e.g. surveillance, bedside reviews), incorporating education on IPC operations, and enhancing connection between ID fellows and IPC non-physician team members.

We partnered an ID fellow and an IPC program manager to lead development of the new IPC curriculum. The designed curriculum consists of 3 weeks separated throughout fellowship focusing on: 1) IPC core concepts, and 2) IPC operational skills. Weeks 1 and 2 are a primer on IPC as a field including emphasis on the multidisciplinary roles involved. Week 3 focuses on the role of IPC within overall hospital operations. An optional 4^th^ week is available for those pursuing a career in IPC.

**Results:**

We have implemented 2 weeks of the new curriculum. The post-implementation feedback is still in progress as only 2 of the 3 weeks has been implemented. Initial feedback highlighted the interactive nature of the curriculum and organized delivery. Additionally, feedback from IPC team members highlighted the improved communication with fellows and better understanding of a fellow’s role.

IPC Curriculum Overview

An overview of the newly developed IPC curriculum for pediatric ID fellows. Week 1 and 2 are focused on IPC fundamentals, Week 3 is dedicated to operational knowledge and skill, and an optional week 4 exists for those particularly interested in IPC as a career.

IPC Foundations "Passport"

This is an example of the passport tool for tracking experiences during the IPC rotation weeks. This is used by both the fellow and the IPC division to help create shared awareness of a fellow's experiences and better identify any gaps.

Key Elements of Operations Week.

This schematic illustrates the key components of week 3 (Operations) of the IPC curriculum. The 3 main elements include collaborating on an IPC-related project, didactics of IPC emergencies (with opportunity to answer IPC calls with infection preventionists and IPC medical directors), and a focus operational skills essential to being a leader within IPC.

**Conclusion:**

Through collaboration between the ID fellowship program and IPC, we redesigned the IPC curriculum for fellows. While implementation of the curriculum is still in progress with ongoing plans for evaluation, we have demonstrated success in engaging a multidisciplinary team to develop a curriculum for ID fellows focused on an operational (rather than strictly content) field.

**Disclosures:**

**All Authors**: No reported disclosures.

